# Interaction preferences across protein-protein interfaces of obligatory and non-obligatory components are different

**DOI:** 10.1186/1472-6807-5-15

**Published:** 2005-08-16

**Authors:** Subhajyoti De, O Krishnadev, N Srinivasan, N Rekha

**Affiliations:** 1 Department of Biotechnology, Indian Institute of Technology, Kharagpur, 721 302, India; 2 Molecular Biophysics Unit, Indian Institute of Science, Bangalore 560 012, India

## Abstract

**Background:**

A polypeptide chain of a protein-protein complex is said to be obligatory if it is bound to another chain throughout its functional lifetime. Such a chain might not adopt the native fold in the unbound form. A non-obligatory polypeptide chain associates with another chain and dissociates upon molecular stimulus. Although conformational changes at the interaction interface are expected, the overall 3-D structure of the non-obligatory chain is unaltered. The present study focuses on protein-protein complexes to understand further the differences between obligatory and non-obligatory interfaces.

**Results:**

A non-obligatory chain in a complex of known 3-D structure is recognized by its stable existence with same fold in the bound and unbound forms. On the contrary, an obligatory chain is detected by its existence only in the bound form with no evidence for the native-like fold of the chain in the unbound form. Various interfacial properties of a large number of complexes of known 3-D structures thus classified are comparatively analyzed with an aim to identify structural descriptors that distinguish these two types of interfaces. We report that the interaction patterns across the interfaces of obligatory and non-obligatory components are different and contacts made by obligatory chains are predominantly non-polar. The obligatory chains have a higher number of contacts per interface (20 ± 14 contacts per interface) than non-obligatory chains (13 ± 6 contacts per interface). The involvement of main chain atoms is higher in the case of obligatory chains (16.9 %) compared to non-obligatory chains (11.2 %). The β-sheet formation across the subunits is observed only among obligatory protein chains in the dataset. Apart from these, other features like residue preferences and interface area produce marginal differences and they may be considered collectively while distinguishing the two types of interfaces.

**Conclusion:**

These results can be useful in distinguishing the two types of interfaces observed in structures determined in large-scale in the structural genomics initiatives, especially for those multi-component protein assemblies for which the biochemical characterization is incomplete.

## Background

Proteins interact with other proteins and bring about myriad of molecular activities in the cell. Interacting proteins are known to play key roles in almost all cellular and biological processes such as metabolism, endocrine, exocrine and paracrine signaling, protein synthesis and trafficking [1]. With the availability of genomic data in abundance, it is important to conceive protein-protein interactions structurally and be able to predict those proteins that might potentially bind to each other.

Many protein-protein interfaces are permanent and the polypeptide chains remain bound to each other throughout their functional lifetime. The complex between β and γ-subunits of hetero-trimeric G-proteins forms a classical example. There are several examples of dimeric enzymes, such as triose phosphate isomerase, in which the interface formed between the subunits can be considered permanent. We refer such interfaces and subunits as obligatory.

On the contrary, there also exist protein-protein complexes that are transiently formed and the proteins detach from each other in specific biological situations. The overall structures of these proteins are stable in the unbound form and as they bind to each other. Conformational changes are possible in one or both the proteins as they switch between bound and unbound forms. There are several known examples of this kind. One of the examples is the complex formed between cyclin and cyclin-dependent protein kinase. Such complexes can be deemed as non-obligatory interactions and they act as switches and bring about regulation of a number of proteins in the pathway in which they occur.

Physical interaction between proteins is viewed best as interactions between structural domains as a domain is often the minimal module corresponding to a biochemical function. However the interacting domains could arise from the same polypeptide chain or different polypeptide chain. Inter domain interfacial properties, with both the domains arising from the same polypeptide chain, are observed to be intermediate to homodimeric (inter-chain obligatory) and non-obligatory complexes [2]. Inter chain protein-protein interaction may be formed between two identical chains (Homodimers) or between two different chains (hetero complexes) or it could be a special category of association such as antigen-antibody complexes. Each of the above types of interfaces is ranked based on the chemical and geometrical parameters and it was detected that a single parameter could not be used to definitively distinguish one type of interface from rest of the tertiary surface [3].

The interactions of non-obligatory components can be transient or weaker (compared to interactions between obligatory subunits), although specific. Weak interactions may exist also in the interfaces of those protein homooligomers that are known to exist as both monomer and oligomer at physiological conditions. Transient interactions are those that are more stable and the association or dissociation process requires a molecular trigger. The interfacial property analysis on such a dataset revealed that there exist distinct physicochemical and geometrical properties between these two types of transient complexes [4].

Ofran and Rost [5] classified protein-protein interaction complexes from the protein data bank (PDB) [6] in conjunction with Swissprot [7] into various categories such as the interfaces formed between domains within a polypeptide chain, homo and hetero types of obligatory and non-obligatory complexes. They detected characteristic amino acid compositional preference for each type of interfaces so obtained. Thus, it is generally expected that obligatory and non-obligatory subunits may be characterized by distinct physico-chemical properties.

Bahadur and coworkers [8] analyzed a set of homodimers and reported that the interfacial properties such as the interface area and the hydrophobicity of the interface of these homodimers are distinctly different from that of the protein complexes formed after the subunits fold into tertiary structure.

Attempts have been made to distinguish protein-protein interaction contacts from crystal contacts. It has been noticed that the protein-protein interaction sites are generally larger in surface area than non-specific crystal contacts [9]. This distinction alone however, is not accurate in clearly demarcating the specific and non-specific contacts. In addition to interface area, the conservation of interfacial residues seems to be more robust descriptor to distinguish the crystal contact and specific protein-protein interaction contact [10]. In a recent analysis on a selection of 122 homodimeric protein-protein interaction complexes and 70 protein-protein complexes (representing nonspecific interactions) it has been noticed that the residue propensity and hydropathy along with interfacial area at the contact surface helps in distinguishing the two types of interfaces [11].

Taking lead from the earlier work, the present analysis aims at addressing more complex problem of recognition of the obligatory and non-obligatory complexes from the PDB. Much of the earlier reported analyses relied on datasets derived from hand picked cases from the PDB or are concentrated on homodimeric protein-protein complexes for representation of permanent (obligatory) complexes. We try and overcome both these drawbacks by devising a homology-based method to identify non-obligatory complexes. The current analysis takes advantage of the fact that PDB [6] is a storehouse of all known protein structures and the present multimeric population of proteins in the PDB reveals that there exists a large repertoire of monomeric and oligomeric structures [12]. We have formed a non-redundant data set of obligatory and non-obligatory chains identified by analyzing various protein-protein interaction complexes. The steps involved in the formation of our dataset help us in the identification of homo and hetero protein-protein interaction complexes clearly distinguished as obligatory or non-obligatory. We then analyze various interface properties with an objective to interpret the differences at the interface level that would help in identifying and distinguishing the obligatory and non-obligatory interactions starting from the 3-D structures of protein-protein complexes.

Glaser and co-workers [13] have analyzed residue contact preferences in a dataset of 621 protein-protein complexes. They observe that all types of contacts are observed at the interfaces, especially the hydrophobic contacts. They also report that large interfaces are more abundant in non-polar contacts and small interfaces are abundant in polar contacts. In present study we observe that obligatory and non-obligatory residue contacts across interface together makes up for the general trend. The highest number of contacts between residues across interface that is reported for obligatory homo oligomer is between identical residues [5]. The hetero obligatory complexes and both types of transient oligomers (homo and hetero type) have predominance of polar interactions. Frequency of non-polar contacts reported here are generally lower, when compared to polar contacts [5]. Complementary to earlier studies we not only study the residue-residue interactions across interfaces, we also analyze the local secondary structures involved in interface formation and contribution of main -chain atoms to the interactions.

The rules thus derived can be applied to protein engineering projects aimed at stabilizing otherwise weak interactions as reported in the case of monomeric protein L [14] or destabilize the interactions. This notion regarding the nature of interaction can guide in designing small molecules that might disrupt associations between two proteins and probably have the potential to act as drug molecules. Our results could also help in predicting obligatory or non-obligatory nature of a polypeptide chain that is seen to form protein-protein interactions in the experimentally determined 3-D structures of protein assemblies.

## Results and discussion

The basis of our classification of protein chains as obligatory and non-obligatory relies on the fact that an obligatory chain depends on binding of another chain for maintenance of overall structure, stability and function. Thus one would expect that in the PDB, the three dimensional structures of obligatory chains are deposited with its partner chain. Further, availability of crystal or NMR structure of an obligatory chain not bound to another polypeptide chain is not expected. A simple-minded PSI-Blast [15] based homology search is implemented here to distinguish between the different types of chains.

Present dataset is derived from a recent release of PDB that contained tertiary structure for 47,550 chains. This set was subjected to a number of filters to obtain a dataset of obligatory and non-obligatory protein complexes.

A chain and its close homologues with no independent (unbound) tertiary structures is deemed obligatory chain and the region on its tertiary surface that makes the contact with the other chain in the complex is said to be obligatory interface. Those complexed chains that have clear sequence similarity to a monomeric structure are deemed non-obligatory chains and the interface such a chain makes with its partner chain in the complexed state is considered as non-obligatory. We have manually scrutinized initial lists obtained, by these considerations, by referring to the literature and removed erroneous and doubtful cases from the final dataset for analysis. The final dataset contains 82 obligatory chain entries and 30 non-obligatory chain entries. These examples are listed in Table 1. While this list is unlikely to be comprehensive, since we have removed doubtful cases, the entries in the final dataset are often either clearly obligatory or non-obligatory. All analysis presented here are performed on the observed protein-protein interfaces of each of the obligatory or non-obligatory chain and its partner chain. The interfaces are defined by identifying those residues that show significant change in the solvent accessibility upon complex formation.

The stringent criterion identifies only those interfacial residues that show large changes in accessibility on complex formation and are located at the center of the interaction patch (which is confirmed by manual inspection), and are shielded from the solvent molecules in the bound state.

The generous criterion, on the other hand, is a lenient measure and identifies all the residues that show variation in accessibility, however small it is, between complexed and free forms. This criterion identifies all the residues in the interface. The residues that are located in the central region of the interface as well as the residues in the surrounding region (periphery of the interface patch) are also picked up by this scheme of defining interface. The interfacial residues at the periphery of the patch are not completely buried and remain solvated even in the bound form.

### Residue propensity

**Figure 1:**
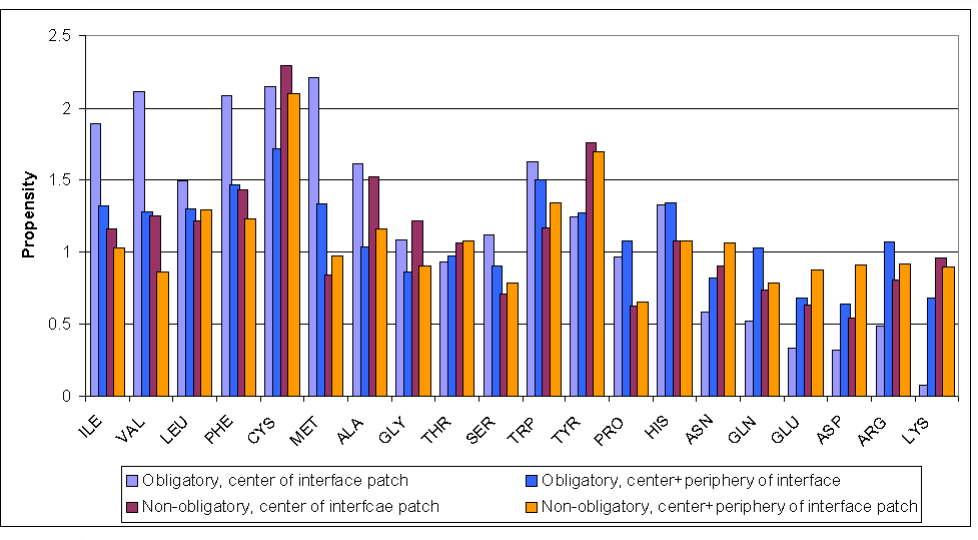
Plot showing the residue propensity in obligatory and non-obligatory interfaces. Propensities of the residues in the entire interface as well as at the core of the interface are shown.

Propensities of individual residues to exist in the protein-protein interface are calculated for both obligatory and nonobligatory interfaces for the entire interface as well as for core of the interfaces and the results are shown in figure 1. The propensity values aid in identifying the residues commonly occurring at the interface and also reveal the chemical nature of the interfaces. A propensity value greater than 1 indicates that the frequency of occurrence of that amino acid at the interface is higher than rest of the domain surface.

The comparative graph of residue propensities for obligatory and nonobligatory interfaces plotted for core and entire interface shows that center of the obligatory interfaces have high propensity for mainly nonpolar residues such as Ile and Met although other hydrophobic residues are also preferred. Other than a slight preference for Trp, Ser and His, no other polar residue is found to occur preferentially at the center of obligatory interface patch. This observation can be explained by considering the residue-residue contacts and are analyzed as described in a later section. These residues appear to strengthen binding at the interface by aromatic and polar interactions.

The core of non-obligatory interface has a large frequency of occurrence of short non-polar residues and aromatic residues such as Tyr. Interestingly, polar residues such as Arg and Gln show a slight, preference for occurrence at the core of the non-obligatory interface. High propensity of short nonpolar residues such as Leu, Val can provide the necessary flexibility in transient interaction in case of non-obligatory interfaces and polar residues can bring about the necessary strength and specificity. A slight preference for Pro to occur at non-obligatory center is noted. Pro is a structurally constrained residue, and shows feeble participation in regular secondary structures, and is chemically non-polar, contributing to hydrophobic interaction in protein structures. Thus Pro is expected to favor non-obligatory interfaces, providing irregular regions and turns, which is a hallmark of non-obligatory interfaces.

Trp is seen to be present in both types of interfaces, both at the center and at the periphery. The relative occurrence of Trp in proteins is small [16], and it is also known to be well-conserved [17]. Evidence suggests that Trp is the most favored residue as the interaction hot spots [18]. Hot spot residues are those residues that contribute maximally to the binding energy. It can thus be postulated that it plays a role in domain-domain recognition in transient interactions apart from balancing the positively charged Arg by cation-π interaction [13]. This could explain the larger propensity of Trp in non-obligatory interfaces, where recognition of the interface patches becomes essential for association and dissociation steps during the course of the functionality of the protein. In obligatory interfaces, Trp could be assisting in interface formation by virtue of burial of its large surface area upon complex formation.

It is surprising to note the high propensity of occurrence of Cys in interfaces. Cys may be considered a weakly polar residue when it is not involved in disulfide formation. Compared to Lys, Arg has higher probability of occurrence at interface. The acidic residues are not preferred at the interface in comparison to domain surface. The detailed picture of residue contacts observed in the dataset is discussed in a later section.

Polar residues are primarily picked up for both obligatory and nonobligatory interfaces, when both the center and periphery of the interface (entire interface) is considered. From the figure 1 it becomes clear that polar residues such as Thr and Tyr show roughly equal tendency to occur in interfaces of both types- obligatory and non-obligatory. It can be suggested that the polar residues are more frequent at the periphery of the interfaces than at the center. Also, since polar interactions are directional they may play a role in maintaining specificity of the interaction.

**Figure 2:**
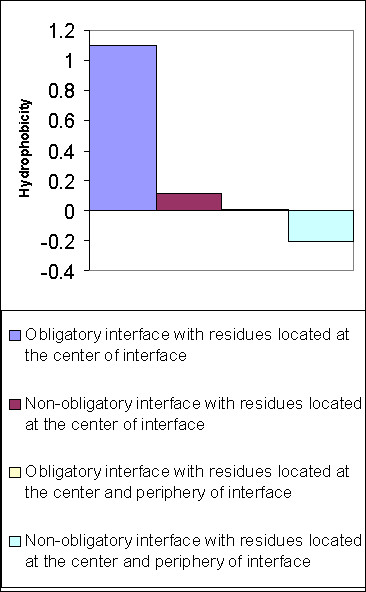
Hydropathy plot for the interface patch. The figure shows hydropathy plot for both obligatory and non-obligatory interfaces and for the residues in the core of the interface and in entire interface.

The analysis of overall hydrophobicity of the interface for the core and entire interface (as plotted in figure 2) reveals that the centers of obligatory and non-obligatory interfaces are predominantly apolar. Additionally, the periphery of both the types of interfaces is more polar in nature as compared to center. Similar observation was made, in an earlier analysis on a general dataset of protein complexes [19]. This could result in favorable interactions of the residues at the periphery of the interface with the solvent. Interestingly, the non-obligatory interfaces are more polar in nature as compared to obligatory interfaces when the center and periphery of the interfaces is compared, probably because the interfacial residues interact with solvent when the non-obligatory pairs exist as tertiary structures not bound to each other.

### Residue contacts at the interfaces

The residue contact analysis is aimed at identifying the pairing pattern of the local regions and interacting residues across the interface. Only the contacts made by the obligatory or non-obligatory chain to the interface formation is considered here. The contribution of partner chain is not considered here unless, it is also present in the dataset of either obligatory or non-obligatory class. The contacts made by each chemical group of residue from the obligatory or non-obligatory chain with another chemical group from the partner chain are considered for the contact matrix generation. Both main-chain and side-chain atoms are considered for the analysis. The interactions were broadly classified into polar and nonpolar. Inter-subunit disulphide links are rare and we observe only three disulphide bridges across the subunits of obligatory chains in the present dataset.

**Figure 3:**
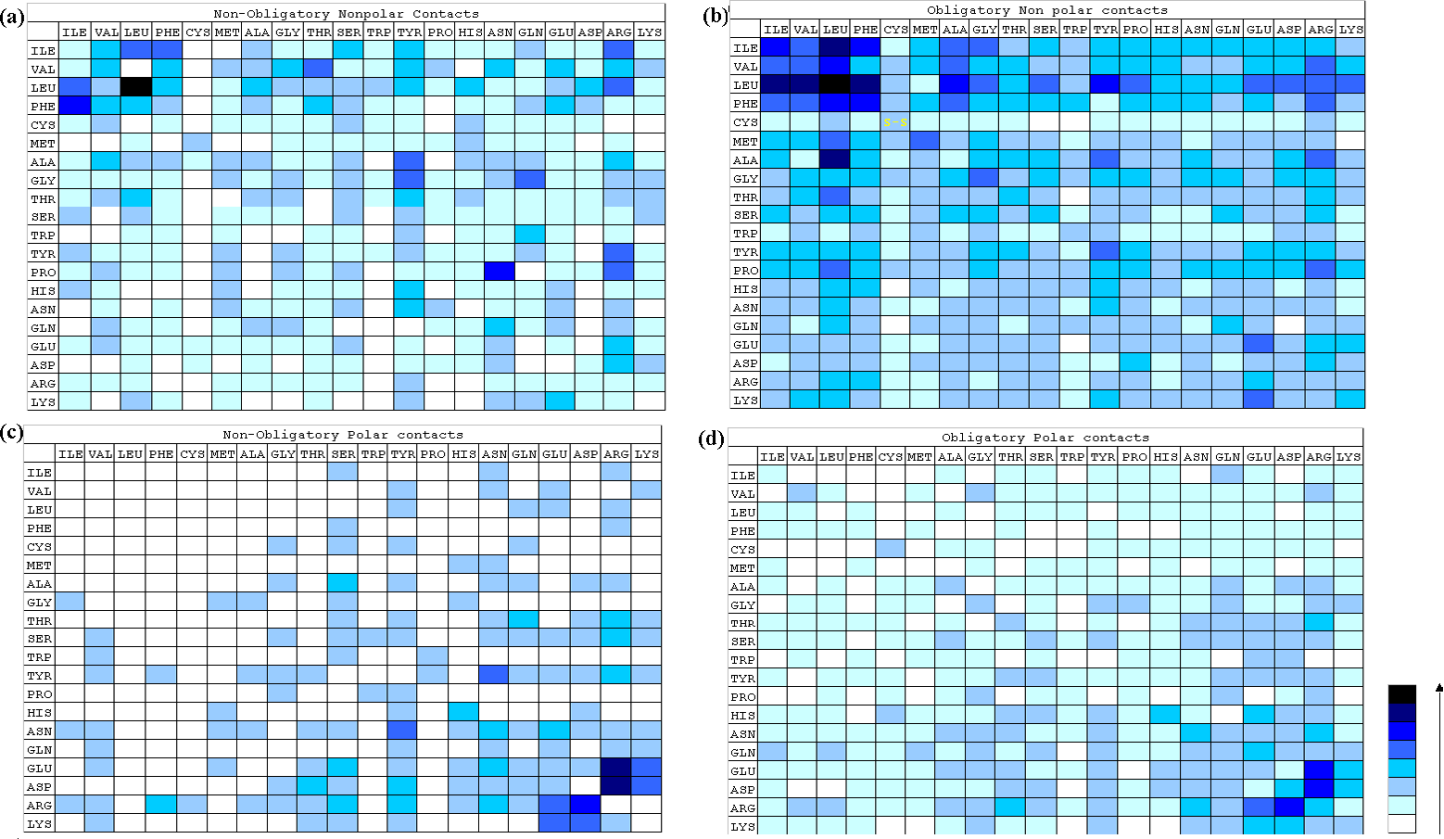
Residue contact matrix showing the frequency of the contact between two residues at the interface. a: Non-obligatory non-polar interactions, b: obligatory non-polar interactions c: non-obligatory polar interactions d: obligatory polar interactions. The residues contributed by the obligatory or non-obligatory chain of the complex is represented in rows. The residues from the partner chain (for which the assignment of obligatory or non-obligatory is ambiguous) is shown in columns. Colour gradation: white- No interaction; Cyan to black- increasing gradation of interaction with normalised frequency varying between 0–14 in discrete steps.

The results are summarized in figure 3. All the interactions observed are normalized and are color coded, with lighter shades indicating fewer contacts observed and darker shades indicating larger number of contacts observed. The values of the observed contacts between different residues that are colour coded vary between 0 and 14 and colour intensity increases in discrete steps (white color indicates no interaction). These values represented in the matrix are the normalised values of the observed number contacts for the obligatory and non-obligatory chain. (*Materials and Methods *section covers the details of calculation of the values represented in the matrices). The extent of non-polar contacts observed for non-obligatory examples is shown in figure 3a and that observed for obligatory cases in depicted in figure 3b. Similarly, the extent of polar contacts observed for non-obligatory examples is shown in figure 3c and that observed for obligatory cases in depicted in figure 3d. The residues that contribute to the contacts made by obligatory or non-obligatory chain is shown along the rows, and the partner chain residues are shown along the columns.

Comparison of apolar contacts (as shown in figure 3a and 3b) between obligatory and non-obligatory complexes reveal that the contact frequency is marginally higher in the case of obligatory complexes. The cumulative values of normalised contacts are 1229 and 1215 respectively for obligatory and non-obligatory chains. This observation is suggestive of the fact that the obligatory interfaces are dominated by apolar contacts. The cumulative contribution of non-polar residues to the contacts in obligatory is 49.3% of the total apolar contacts, while the contribution of non-polar residues to in contacts made across interface in non-obligatory complexes is 42% of the total apolar contacts. The normalised average contacts made by non-polar residues like Leu, and Phe is higher in case of obligatory chains (8.73 and 5.3 contacts respectively) as compared to non-obligatory chains (6.29 and 4.4 contacts respectively). In case of non-obligatory chains, weakly polar residue such as Cys and Thr contribute to non-polar contacts. The average contact values for Cys and Thr are 1.87 and 3.59 contacts respectively for non-obligatory chains and 0.68 and 2.37 contacts respectively for obligatory chains. Though an isolated van der Waal's contact is weak, large numbers of such contacts can have a collective effect and could contribute to large binding energies. Similar effect could also contribute to stable bound states of the obligatory interfaces.

The residue contact matrix for polar interactions across interface for non-obligatory and obligatory interfaces (as shown in figure 3c and 3d) reveals that the polar contacts are formed mainly between side chains of polar or charged residues for both obligatory and nonobligatory interfaces. However, the main chain amide and carbonyl groups are also seen to contribute to some of the polar interactions shown in the matrix.

The polar interactions that the non-obligatory chains make with their partner chain are represented in figure 3c. We observe that larger number of polar contacts are made by the non-obligatory chain when compared to obligatory chain. The cumulative numbers of normalised contacts are 378 and 391 for obligatory and non-obligatory chains respectively. The polar groups of polar side chains primarily contribute to the contacts in the non-obligatory chain. For example, on an average, 2.62 and 2.39 contacts are made by acidic residues, Glu and Asp present in interfaces of non-obligatory chains. However, Glu and Asp makes only 1.69 and 1.63 average contacts respectively in interaction mediated by obligatory chains. Polar contacts observed in obligatory chains are more distributed, in terms of residue involvement, when compared to contribution from non-obligatory chains. Interestingly, polar atoms in the main chain of the residues mediate a large section of the obligatory polar contacts.

Comparing all the contact matrices in figure 3, we observe that obligatory interactions show extensive apolar contacts and the polar contacts in the obligatory interfaces are largely mediated by main chain atoms. Polar atoms of the polar residues on the other hand mediate non-obligatory polar contacts.

Interfacial residue propensity of Thr is slightly higher for non-obligatory chains, and the interfacial propensity of Cys is higher for non-obligatory chains when compared to obligatory chains (as can be visualised from figure 1). Both these residues can be considered as weakly polar and on dissociation of the non-obligatory chains, it would be favorable for them to interact with solvent. Thus nature has carefully designed the non-obligatory interface, with precise balance of polar, non-polar and weakly polar residues.

### Involvement of Arg, Tyr and Cys in contacts at interface

Interestingly, the involvement of Arg in polar and nonpolar interaction in both non-obligatory and obligatory interfaces is significant. The long nonpolar part of the side-chain of Arg is observed to interact with large nonpolar residues. The interaction of Arg with aromatic side chains indicates the involvement of cation-π interaction. Propensity analysis indicates that aromatic resides are found to be abundant in interfaces, and specifically Tyr is frequent in non-obligatory interfaces.

Tyr is a special case, as it can contribute both to aromatic and polar interactions. On the other hand, the center of nonobligatory interface consists of both polar and nonpolar residues. An interesting observation is the high propensity of Arg at the center of non-obligatory interface. Probably, the ability of Arg to take part in polar as well as in nonpolar interaction using its long nonpolar side chain or by cation-π interaction with phenyl ring of aromatic residues assist in formation of nonobligatory interface significantly. It favors Arg to interact with the solvent (water) in unbound state, and on the other hand, in the complex form, Arg can potentially interact with all types of polar, non-polar or aromatic residues by virtue of the carbon atoms in the side chain, and the positively charged guanidino group.

Similary, it is surprising to find high prevalence of Cys at the interfaces. Cys may be considered as weakly polar if it is not involved in the formation of disulfide. From the figure 3a, we infer that Cys does participate in apolar contacts. Interaction of sulphur with aromatic groups in proteins has been reported. [20,21]. Such a possibility of Cys interacting with aromatic ring systems is raised (P. Chakrabarthy, personal communication).

### Secondary structure analysis

The secondary structures at the interfaces are classified as helix (H), β-strand (E) and others such as turns and loops both collectively represented (T). The conformation of the interfacial residues contributed by both obligatory and non-obligatory chains falls into all the three above-mentioned classes.

In obligatory interfaces 45.8% of total interface residues were involved in helix-helix interaction while only 31.3% of total interface residues are involved in helix-helix interactions in case of non-obligatory interfaces. Thus, interactions between two helices were noticed in both obligatory and non-obligatory types of complexes.

Non-obligatory interfaces have higher involvement of irregular secondary structural region (either defined as turns 'T' or as unassignable). 12 and 37.4% of the total interface residues in case of non-obligatory and 9.1% and 16.9% of the total interface residues in case of obligatory complexes are observed to form turns or irregular secondary structures. This probably provides the necessary flexibility to the interface to favor the interacting subunits to dissociate under appropriate conditions.

While examining the examples of non-obligatory interactions we found no instance of β-sheet formation across the two subunits at the interface. On the other hand in the case of obligatory interactions, out of the 28.3% of total interfacial residues participating in formation of strands 3.4% of it were detected to form inter-subunit β-sheet. Only 19.3% of total interfacial residues from non-obligatory class contribute to strands at interface. Such β-sheet formation across interface makes the complex formed very stable, and in such examples, polar contacts are the driving force in interface formation, and non-polar contacts are less prominent.

Hence it can be inferred that the involvement of secondary structures elements for interface formation is more characteristic of obligatory surfaces (P value < 0.05 for the involvement of helix as well as β-sheet at the interface).

The interaction between the secondary structures especially the β-sheet formation is mediated by the interaction between the main chain atoms. We quantified the main chain-main chain (MC-MC), main chain-side chain (MC-SC), side chain-side chain interactions (SC-SC) in both cases of obligatory and nonobligatory interactions. The extent of MC-MC (16.9% of total contacts in case of obligatory and 11.2% of total contacts in case of non-obligatory) is the most distinguishing between two types of interfaces when contacts are considered at atomic level. The values obtained for MC-SC (about 42.6% of total contacts in case of obligatory and 49.3% of total contacts in case of non-obligatory) and SC-SC (40.5% of total contacts in case of obligatory and 39.6% of non-obligatory) are mostly comparable. However, we note that the main chain involvement is clearly higher for obligatory examples (P value < 0.15 using t-test).

### Interface area distribution

**Figure 4:**
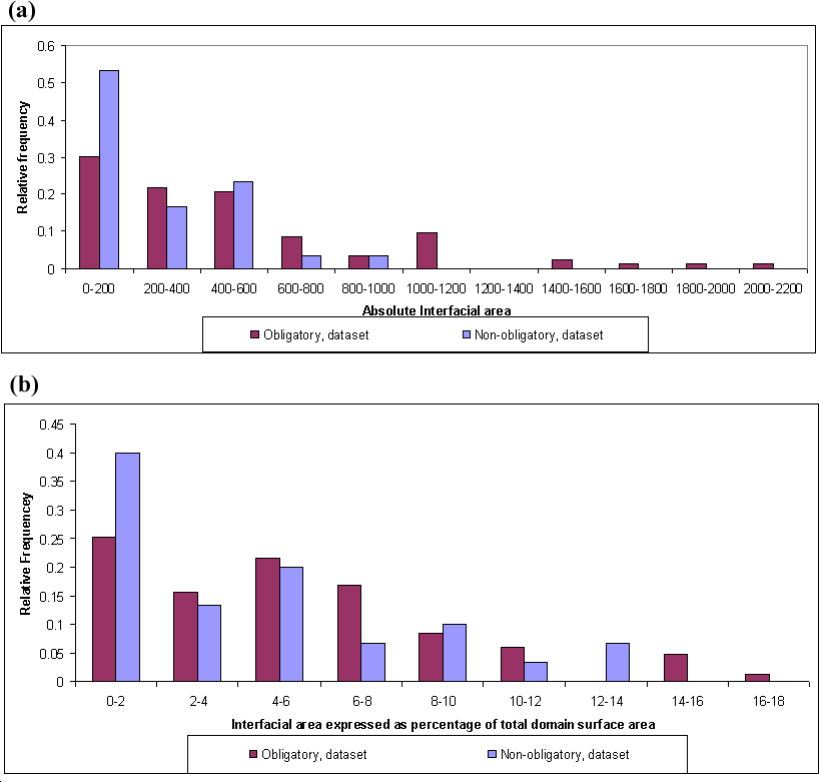
a: Distribution of the absolute interface area for obligatory and non-obligatory protein complexes in the general dataset. b: Distribution of percentage occupancy of interface for obligatory and non obligatory complexes in the general dataset.

Interface areas are calculated for both obligatory and non-obligatory protein complexes and the results are summarized in figure 4. The plot presented in figure 4a is the frequency of absolute interface areas for both types of interfaces. The average interface area in case of obligatory interfaces is 492.74 Å^2 ^and in the case of non-obligatory complexes it is 279.55 Å^2^. From the plot given in Figure 4a, we observe that the obligatory interface has a higher mean value and a broader distribution in raw interfacial area. This implies that the nonobligatory interfacial areas are generally smaller (P value < 0.05 using t-test) and this translates to less strong interaction that might help in making the interaction transient. This point is further validated by considering the average number of contacts per interface in the two cases. The obligatory complexes make 20 contacts per chain on an average whereas non-obligatory complexes make 13 contacts per chain. The number of contacts per chain can be taken as a rough measure of the strength of interaction. In the dataset derived in this work, the number of contacts in obligatory interfaces is shown to be significantly different than the average number of contacts made by non-obligatory interfaces (P value <0.05 using modified t-test). It must be pointed out that, even though the number of contacts seen is different in the two cases, the contact density is similar (0.08 contacts / Å^2 ^in case of obligatory complexes and 0.06 contacts / Å^2 ^in the case of non-obligatory complexes). This means that the interfacial packing density is not different in the two cases.

Viewing interfacial areas as a fraction of total domain surface area, we observe 41% of the non-obligatory interfaces occupies ≤ 2% area of the domain surfaces. However, in the cases of obligatory interfaces, there is an even distribution of examples between 0–6% area of domain surface, with 80% of examples in this range (figure 4b). The surface areas of the domains considered here are large, hence, we observe that the interface occupation on tertiary domain surface is small. However, their absolute areas in these are mostly comparable with the other obligatory interfaces in (Å^2^).

Among the cases of obligatory complexes, there are instances of huge multi-subunit protein machinery like proteosome (1ryp_1), where single interface (formed between two subunits of multi subunit complex) occupancy is very low on the total domain surface. Most of these examples correspond to those proteins that have small domain surface area and part of a large multi-domain complex.

### Analysis of the topology of the interfaces

Shape complementarity of the interface using SC-program of Lawrence and Colman [22] for the pair of interacting proteins. Overall both type of interfaces showed a robust clustering of shape complementarity value within a range of 0.6 to 0.8. This implies that the geometrical complementarity at the interfaces of both types of complexes is similar. The average shape complementarity value for non-obligatory interfaces was 0.649 while it is 0.686 for obligatory interfaces. Thus it appears that overall the obligatory interfaces have slightly better shape complementarity though the difference between the obligatory and non-obligatory types of interfaces is very small (P value > 0.4 using t-test).

## Conclusion

We have arrived at a set of protein complexes from the PDB, classified in broad terms as belonging to obligatory or non-obligatory categories using a simple sequence analysis based procedure. The assignment of obligatory or non-obligatory nature is restricted to the chain level and the interaction interface of this chain.

Present analysis is attempted to find the distinguishing features of the two types of interfaces. While nonpolar contacts dominate the interaction interfaces, especially the obligatory interactions, the polar interactions are also observed in interfaces. The polar interactions are mediated by hydrophilic sidechains in the cases of non-obligatory interactions probably provides favorable binding energies, and also helps to stabilize the tertiary structure when the complex dissociates. On the other hand main chain polar groups have a substantial representation in the obligatory interfaces.

Non-obligatory or transient interactions are likely to be characterized by optimal binding energy, so that the complex can be disassembled into its constituent elements upon a molecular stimulus. Additionally, the interacting subunits of the non-obligatory complex interacts with polar solvent in the uncomplexed form and hence the non-obligatory interfaces are less hydrophobic. While we have not made an explicit analysis of role of water in protein-protein interfaces, it is expected that water can interact favorably with polar amino acid residues at the interface. Indeed a water molecule is observed to contribute to polar contacts between two macromolecules [23].

The β-sheet formation across the interface is a feature seen in the case of obligatory interfaces. However, none of the non-obligatory cases analyzed here has this feature. The main chain contribution to the interface is clearly more prominent in obligatory interfaces.

Covalent association in the form of disulphide bridges between the subunits is a feature of obligatory complexes. The covalent associations make the protein-protein interaction permanent. There are exceptions to this rule, like the case of the type II ribosome inactivating plant toxins where the toxic chain is covalently linked *via *a disulphide bond to a carrier lectin moiety. The disulphide bond reduction and release of toxin is an essential step for biological activity of the protein. Here although the plant toxin- lectin association is non-obligatory, a covalent association of the subunits is observed making the complex formed between the two proteins extremely stable and are dissociated only under specialized conditions.

The key feature of obligatory type of interfaces is its stability of association. This stability is achieved in a number of systems in diverse manners. For example, if extensive β-sheet formation is the strategy adopted to form obligatory interface in a certain protein, for example the lectins, then it is observed that the interfaces can be more polar than the generally observed trend, and the protein-protein interaction is sensitive to pH variations [24]. In such cases, the residue propensities do not obey the general rules. For this reason, the test data set considered here has marked deviations in the residue propensities compared to the original dataset.

Thus from the carefully chosen set of obligatory and non-obligatory complexes, the analysis shows distinction between obligatory and non-obligatory interfaces in terms of some of the features such as patterns of interaction across the interfaces. There is a clear trend for the obligatory interfaces to be larger in area, the center of obligatory interface to be non-polar, and to involve stable secondary structural elements across the interface. Since the variations between different types of interfaces are subtle, a single feature cannot be reliably used to predict different types of complexes. However a cumulative effect of all these features can aid recognizing obligatory and non-obligatory interfaces. The results of statistical tests on various features suggest that differences in only some of the features are statistically significant. However our analysis provides an indication of the trend which may be strengthened by the accumulation of more 3-D structures of protein-protein complexes.

Thus, a combination of above said features, when considered concurrently and appropriately weighed can add value to the prediction of obligatory and non-obligatory interaction sites on the tertiary surface. Such an approach is shown to be successful using a test dataset not used in the original analysis.

Association of wide variety of proteins mediates many vital cellular processes. To be able to model the tertiary and quaternary structure from the primary structure is the goal of comparative modelling approaches [12]. Such problems are best addressed by considering the structural information of a homologous protein, since it is observed that protein-protein interaction sites are evolutionarily conserved among close homologues [10,11]. However, in cases where the information on association cannot be directly derived based on homology, the present analysis can aid in determining the nature of the interface. This information about the nature of interface formed gives an indication of the stability of the complex.

The results presented in this paper can also be useful in distinguishing the obligatory and non-obligatory types of interfaces observed in structures determined in large-scale in the structural genomics initiatives, especially for those multi-component protein assemblies for which the biochemical characterization is incomplete.

## Methods

### Dataset generation

The co-ordinate sets of protein structures used in the analysis were extracted from the April 2003 version of PDB . All nucleic acid, hetero-atoms, small peptides (<30aa) and extremely large chains (>1000aa) were excluded from this raw set. All other protein chains were retained and taken for further analysis.

The polypeptide chains in many of the protein-protein complexes could be classified into one of obligatory or non-obligatory subunit. Each entry in the PDB is classified as monomer or multimer depending on number of chains in the structure and by consulting the Protein Quaternary Structure server [9]. Each chain from the monomer data set was searched for homologues in the multimer chain set using single round of PSI-blast run using 0.01 as E-value and 0.001 as inclusion value (h-value) [15]. The protein chains in monomeric set that have homologues having 95% sequence identity over 90% of the monomer chain length is assumed to exist in monomeric form as well. This means that being in the oligomeric state is not mandatory for their structure and functionality. Subsequently their interactions with physically adjacent protein chains within the same protein were considered as nonobligatory interaction. On the contrary, in case of obligatory entries, it was assumed that the state of oligomerisation is essential for the structure and functionality of the subunit. So the protein chains that are not nonobligatory were considered to be probably obligatory and their corresponding interactions with neighboring subunits were also considered obligatory. e.g. in G-protein the β and γ subunits are always bound to each other whereas α-subunit alternate between bound and unbound forms with β,γ subunits depending upon if GTP or GDP is bound to the α-subunit. So we consider α-β interactions are nonobligatory while β-γ binding is an obligatory interaction. The interactions between polypeptide chains within a protein that have no physical contact between them were kept out of consideration.

The polypeptide chain that is considered for search against the monomer dataset is called the representative chain and the chain with which it physically interacts is called the partner chain. The obligatory or non-obligatory nature of the interaction is defined with respect to the interaction of representative chain with its partner in the crystal structure. The obligatory or non-obligatory nature of interaction is restricted to the interactions contributed by the representative chain.

Crystal structures having resolution better than 2.5Å and the best model of NMR structures were considered for the analysis. To avoid redundancy in the dataset, Only those representative structures that had lesser than 25% sequence identity to other structures in the dataset were selected for the analysis. We consulted the PDB-Select [25] that gives a listing of non-redundant collection of PDB structures.

Due to low occurrence of monomeric homologues in the PDB, the entries classified as obligatory interactors purely based on an automatic procedure as described above have high scope for contamination. Hence, we consulted other sources, especially the literature to retain only those entries for which the biologically relevant oligomeric state was clearly and explicitly mentioned. Those cases which are either unclear or lack sufficient information or ambiguous are excluded from the dataset for analysis. We have consulted the published works of Lo Conte and co-workers [26] and Nooren and Thornton [4] and have incorporated entries from their reported datasets in appropriate classes, in case we failed to identify them by our automated procedure.

Antigen-antibody complexes behave as non-obligatory complex in the unbound state; but the binding of the antigen with the antibody is associated with large energy of binding and thus, the interaction can be considered as obligatory. Thus, antigen-antibody complexes are deviant from our definition of obligatory and nonobligatory complexes and hence we did not include antigen-antibody complexes in the present analysis. Similarly, integral membrane proteins have intrinsic amino acid preferences so that they could be accommodated in a hydrophobic environment – the cell membranes. These entries were weeded out from the dataset.

### Defining interface

Inter-chain interfaces were defined following the accessibility changes. A residue is said to be at the core of protein-protein interaction interface if its accessibility values show large variation between exposed (>10%) and buried state (<7%) upon oligomerisation (dimerisation) with the corresponding interacting subunit. This method identifies those residues that are almost fully buried in the complex state and well exposed in the uncomplexed state. Mainly the residues that are at the center of the interface are picked up by this method.

On the other side, the residues at the surface (ASA >7%) which lose solvent-accessible surface area by >1Å^2 ^on oligomerisation, are also considered to be at the interface. This encompasses a larger number of interfacial residues than the number of residues at the core of the interface. Thus using this criterion, the residues are picked up over a broader area or in other words, the residues at the center as well as the periphery of the interface are picked up. The notion of the location of interacting residues in the center and periphery of the interacting region on the surface of protein complex was confirmed by visual inspection on a number of cases.

### Residue propensity

We analyzed residue propensity at the interface to study the preference of the amino acid residue to occur at the interface with respect to the preference of the residue to occur at the surface of protein at the domain level. We referred to the SCOP [27] for the definitions of the composite structural domains of the polypeptide chains.

The residue propensity was defined as -

P(int)_*i *_= N(int)_*i *_/ N(surf)_*i*_

Where, P(int)_*i *_= Propensity of i^th ^amino acid at the interface

N(int)_*i *_= Normalised number of i^th ^amino acid at the interface

N(surf)_*i *_= Normalised number of i^th ^amino acid at the domain surface

Propensities for each of the 20 residue types were calculated for both obligatory and nonobligatory interfaces following both core and entire interface. The propensity analysis reflects the residue preferences of the interfaces and also reveals the chemical nature of the interfaces and kind of interactions present.

The hydrophobic nature of the interfaces was studied by a hydropathy analysis using standard Kyte and Doolittle scale [28]. Hydrophobicity value is calculated as-

Hydrophobicity value = hydrophobicity index * residue propensity.

The analyses were done both for the core and entire interfaces for both obligatory and non-obligatory types of interfaces.

### Residue contacts

One of the objectives of the present analysis is to discern the residue level interactions across the interacting and representative chains. This information is very crucial since it can reveal the nature of interactions and residue pairing preferences across interface.

The residue interactions were classified broadly in 3 groups -

i. Covalent bond forming: Disulphide linkages

ii. Electrostatic and H- bond forming: Polar (salt bridge and H-bond) interactions,

iii. Van der Waal interactions: Nonpolar interaction, interactions involving aromatic ring systems

The polar interactions were considered between uncharged polar as well as charged groups. Hydrogen bonds considered here are formed when the hydrogen associated with nitrogen is shared with acceptor oxygen of carbonyl or carboxyl group. Other hydrogen bonds occur under special geometrical and chemical constraints and are weaker than the above said class. Hence their involvement is not considered here. All types of polar interactions are significant when the N and O are at a distance between 2.4–3.4Å. The apolar interactions were considered to be significant only when the deviation of the sum of the Van der Waal radii of the two atoms is within 1Å distance. Covalent interactions like the disulphide bonds can also be formed at the interface although they are not common at the protein-protein interfaces. The disulphide bonds are considered to exist if the sulphur atoms of the two Cys residues from the interacting chains are at a distance = 2.1 Å. Disulphide linkage provides rigidity and stability in the interaction as compared to electrostatic and Van der Waal interaction.

The inter-chain interacting residue-pairs were picked up on the basis of the kind of interactions they are involved-in. We have classified all the pairwise residue interactions into polar or nonpolar. For a single pair of residues, polar and disulphide interactions were given more priority than Van dar Waal interaction i.e. if a pair of residues present all the types of contacts – polar and non-polar contact, the residue-interaction was considered to be primarily polar and this pair is not considered for its contribution to apolar contacts. The interaction data was classified in polar and nonpolar interactions and presented in the form of 20 × 20 matrices with matrix elements represent the normalized frequency of occurrence of interaction between the residue-pairs. The normalisation was done to account for the disparity in the dataset sizes for obligatory and non-obligatory complexes and also to account for the higher interfacial areas observed in obligatory chains. This normalization ensures that the values obtained for the obligatory and non-obligatory contacts are comparable.

### Interface area

The interface areas for obligatory and nonobligatory interfaces were calculated following core of interface and were represented both in absolute (Å^2^) and as percentage of the total domain surface occupied by the interface. Surface residues were identified if the percent accessibility is >10%. The results were classified according as the fraction of the dataset that have an interface area within a specified range.

### Secondary structures

Secondary structures of the interacting and representative chain were identified using SSTRUC software developed by David Keith Smith (1989, unpublished data) based on the DSSP algorithm [29]. The secondary structures were considered mainly in the broad grouping of Helix (H), B-strand (E) and others (T). A secondary structural element was deemed to be present at the interface of the representative chain, if a secondary structural element contributes at least two residues for interface formation. Similar contributions of helix, strand or loops were calculated for the partner chain also. We then determine the percent of interfacial residues that participate in interactions between the secondary structures across the interface. In the case of extended strands, the possibility of formation of β-sheets across the interface was analyzed by considering potential main chain main chain interactions.

### Shape complementarity

Another geometrical measure, the shape complementarity for interacting chain-pairs was determined by SC program developed by Lawrence and Colman [22]. This program calculates the geometrical packing at the interface between two chains and determines how well the interacting surfaces of the protein complex complement one another. Higher value indicates good geometric complementation, while small values generally indicate bad complementarity.

### Statistical analysis of results

Test for the statistical significance of the results was done using the students t-test. The mean and the variance in the parameter under study of the complexes in obligatory and non-obligatory complexes were calculated. If the variances were found to be significantly similar using the F-test, the normal t-test was used with pooled variances. If the variances were not equal, then a modified t-test with adjusted variances was used. The test statistic in each case was tested at the 0.05 level of significance.

The t-test statistic used to compare the means of absolute interface areas and shape complementarity analysis is given in equation (1) below.

Eq (1)  where, S_p _is given by 

The t-test statistic used for the statistical analysis of main-chain main-chain contact analysis, secondary structure composition analysis and residue contacts per interface is given in equation (2) given below.

Eq(2) 

## Authors' contributions

NS conceived of this study, SD generated the general dataset and carried out the analysis. NR and OK refined the general dataset and carried out the analysis. All four authors have read and approved the manuscript.

## Figures and Tables

**Table 1:** The dataset of obligatory and non-obligatory polypeptide chains identified from the PDB

**LIST OF NON-OBLIGATORY PROTEIN-CHAINS**				
**PDB**	**Representative-chain**	**Partner-chain**	**Resolution**	**Names of proteins**

1dx5	I	M	2.30	Thrombomodulin, Thrombin Heavy Chain
1gua	A	B	2.00	Rap1A, C-Raf1
1efu	A	B	2.50	Elongation Factor Tu, Elongation Factor Ts
1avw	B	A	1.75	Trypsin, Trypsin Inhibitor
1emv	A	B	1.70	Immunity Protein Im9, Colicin E9
1ay7	B	A	1.70	Barstar, Guanyl-Specific Ribonuclease Sa
1stf	E	I	2.37	Papain (Cys 25 Carboxymethylated), Papain (Cys 25 Carboxymethylated)
3eza	B	A	NMR	Histidine-Containing Phosphocarrier Protein H, Phosphotransferase System, Enzyme I
1ggr	B	A	NMR	Phosphocarrier Protein Hpr, Pts System, Glucose-Specific Iia Component
1pyt	B	A	2.35	Procarboxypeptidase A, Procarboxypeptidase A
1pyt	B	D	2.35	Procarboxypeptidase A, Chymotrypsinogen C
1fle	I	E	1.90	Elafin, Elastase
1sgp	I	E	1.40	Turkey Ovomucoid Inhibitor, Streptomyces Griseus Proteinase B
1ycs	A	B	2.20	P53, P53
1ycs	B	A	2.20	P53, P53
1efn	A	B	2.50	Fyn Tyrosine Kinase, Hiv-1 Nef Protein
1efn	B	A	2.50	HIV-1 Nef Protein, Fyn Tyrosine Kinase
1tx4	A	B	1.65	P50-Rho GAP, Transforming protein rhoa
1tx4	B	A	1.65	Transforming protein Rhoa, P50-Rho GAP
1a2k	C	A	2.50	Nuclear Transport Factor 2, Nuclear Transport Factor 2
1fin	A	B	2.30	Cyclin-Dependent Kinase 2, Cyclin A
1fin	B	A	2.30	Cyclin A, Cyclin-Dependent Kinase 2
1ak4	A	C	2.36	Cyclophilin A, Hiv-1Capsid
1ak4	C	A	2.36	Hiv-1Capsid, Cyclophilin A
1dhk	A	B	1.85	Porcine Pancreatic Alpha-Amylase, Bean Lectin-Like Inhibitor
1gla	F	G	2.60	Glycerol Kinase, Factor III Glc
1ydr	E	I	2.20	C-Amp-Dependent Protein Kinase, Protein Kinase Inhibitor Peptide
2pcc	A	B	2.30	Cytochrome C Peroxidase, Cytochrome C
2pcc	B	A	2.30	Cytochrome C, Cytochrome C Peroxidase
1d2z	A	B	2.00	Death Domain Of Pelle, Death Domain Of Tube
				
**LIST OF OBLIGATORY PROTEIN-CHAINS**				
**PDB**	**Representative chain**	**Partner chain**	**Resolution**	**Names of proteins**

1bmq	B	A	2.50	Interleukin-1 Beta Convertase, Interleukin-1 Beta Convertase
1qdl	B	A	2.50	Anthranilate Synthase (Trpg-Subunit), Anthranilate Synthase (Trpe-Subunit)
1poi	B	A	2.50	Glutaconate Coenzyme A-Transferase, Glutaconate Coenzyme A-Transferase
1ihf	B	A	2.50	Integration Host Factor, Integration Host Factor
1poi	B	C	2.50	Glutaconate Coenzyme A-Transferase, Glutaconate Coenzyme A-Transferase
1bcr	B	A	2.50	Serine Carboxypeptidase II, Serine Carboxypeptidase II
1wdc	A	B	2.00	Scallop Myosin, Scallop Myosin
1wdc	A	C	2.00	Scallop Myosin, Scallop Myosin
1ryp	1	I	1.90	20S Proteasome, 20S Proteasome
1ryp	1	J	1.90	20S Proteasome, 20S Proteasome
1ryp	1	S	1.90	20S Proteasome, 20S Proteasome
1ryp	1	T	1.90	20S Proteasome, 20S Proteasome
1ryp	1	Z	1.90	20S Proteasome, 20S Proteasome
1ryp	1	2	1.90	20S Proteasome, 20S Proteasome
1kve	A	B	1.80	Smk Toxin, Smk Toxin
1luc	A	B	1.50	Bacterial Luciferase, Bacterial Luciferase
1fm0	E	D	1.45	Molybdopterin Convertin Factor, Subunit 2, Molybdopterin Convertin Factor, Subunit 1
1fm0	D	E	1.45	Molybdopterin Convertin Factor, Subunit 1, Molybdopterin Convertin Factor, Subunit 2
1h2r	S	L	1.40	Periplasmic [Nife] Hydrogenase Small Subunit, [Nife] Hydrogenase Large Subunit
1h2r	L	S	1.40	Periplasmic [Nife] Hydrogenase Large Subunit, Periplasmic [Nife] Hydrogenase Small Subunit
1svf	C	D	1.40	Fusion Glycoprotein, Fusion Glycoprotein
1svf	B	A	1.40	Fusion Glycoprotein, Fusion Glycoprotein
1hbn	B	C	1.16	Methyl-Coenzyme M Reductase I Beta Subunit, Methyl-Coenzyme M Reductase I Gamma Subunit
1ycp	K	J	2.50	Epsilon Thrombin, Epsilon Thrombin
1hfe	S	L	1.60	Fe-Only Hydrogenase (Smaller Subunit), Fe-Only Hydrogenase (Larger Subunit)
1f95	A	B	NMR	Dynein, Dynein
1a2k	A	B	2.50	Nuclear Transport Factor 2, Nuclear Transport Factor 2
2thi	A	B	2.50	Thiaminase I, Thiaminase I
1e5d	A	B	2.50	Rubredoxin: Oxygen Oxidoreductase, Rubredoxin: Oxygen Oxidoreductase
1b55	B	A	2.40	Tyrosine-Protein Kinase Btk, Tyrosine-Protein Kinase Btk
3nos	A	B	2.40	Endothelial Nitric-Oxide Synthase, Endothelial Nitric-Oxide Synthase
1qfx	A	B	2.40	pH 2.5 Acid Phosphatase, pH 2.5 Acid Phosphatase
2tmk	A	B	2.40	Thymidylate Kinase, Thymidylate Kinase
1f37	A	B	2.30	Ferredoxin [2Fe-2S], Ferredoxin [2Fe-2S]
1qdn	A	B	2.30	N-Ethylmaleimide Sensitive Fusion Protein (N), N- Ethylmaleimide Sensitive Fusion Protein (N)
1dcp	A	B	2.30	Dcoh, Dcoh
1dcp	A	C	2.30	Dcoh, Dcoh
1dcp	A	D	2.30	Dcoh, Dcoh
1dcp	A	E	2.30	Dcoh, Dcoh
1del	B	A	2.20	Deoxynucleoside Monophosphate Kinase, Deoxynucleoside Monophosphate Kinase
1otg	A	B	2.10	5-Carboxymethyl-2-Hydroxymuconate Isomerase, 5-Carboxymethyl-2-Hydroxymuconate Isomerase
1bft	A	B	2.00	Nuclear Factor Nf-Kappa-B P65, Nuclear Factor Nf-Kappa-B P65
1coz	A	B	2.00	Glycerol-3-Phosphate Cytidylyltransferase, Glycerol-3-Phosphate Cytidylyltransferase
1mka	A	B	2.00	Beta-Hydroxydecanoyl Thiol Ester Dehydrase, Beta-Hydroxydecanoyl Thiol Ester Dehydrase
1b66	A	B	1.90	6-Pyruvoyl Tetrahydropterin Synthase, 6-Pyruvoyl Tetrahydropterin Synthase
1otf	A	C	1.90	4-Oxalocrotonate Tautomerase, 4-Oxalocrotonate Tautomerase
1otf	A	D	1.90	4-Oxalocrotonate Tautomerase, 4-Oxalocrotonate Tautomerase
1otf	A	E	1.90	4-Oxalocrotonate Tautomerase, 4-Oxalocrotonate Tautomerase
1j9l	A	B	1.90	Stationary Phase Survival Protein, Stationary Phase Survival Protein
1dfn	A	B	1.90	Defensin HNP-3 – Chain A, Defensin HNP-3 – Chain B
1b93	A	B	1.90	Methylglyoxal Synthase, Methylglyoxal Synthase
1b93	A	C	1.90	Methylglyoxal Synthase, Methylglyoxal Synthase
1ext	B	A	1.85	Tumor Necrosis Factor Receptor, Tumor Necrosis Factor Receptor
1kve	A	C	1.80	Smk Toxin, Smk Toxin
1atl	A	B	1.80	Atrolysin C, Atrolysin C
1d2v	A	B	1.75	Myeloperoxidase, Myeloperoxidase
1tvx	B	A	1.75	Neutrophil Activating Peptide 2 Variant, Neutrophil Activating Peptide 2 Variant
1tvx	B	C	1.75	Neutrophil Activating Peptide 2 Variant, Neutrophil Activating Peptide 2 Variant
6gsv	A	B	1.75	Mu Class Glutathione S-Transferase Of Isoenz, Mu Class Glutathione S-Transferase Of Isoenz
1qsg	G	E	1.75	Enoyl-Reductase, Enoyl-Reductase
1qsg	G	F	1.75	Enoyl-Reductase, Enoyl-Reductase
1qsg	G	H	1.75	Enoyl-Reductase, Enoyl-Reductase
1a2z	A	B	1.73	Pyrrolidone Carboxyl Peptidase, Pyrrolidone Carboxyl Peptidase
1a2z	A	D	1.73	Pyrrolidone Carboxyl Peptidase, Pyrrolidone Carboxyl Peptidase
1mjh	A	B	1.70	ATP-Binding Domain Of Protein Mj0577, ATP-Binding Domain Of Protein Mj0577
1dqi	A	B	1.70	Superoxide Reductase, Superoxide Reductase
1dqi	A	D	1.70	Superoxide Reductase, Superoxide Reductase
1jqc	B	A	1.61	Protein R2 Of Ribonucleotide Reductase, Protein R2 Of Ribonucleotide Reductase
1f74	A	C	1.60	N-Acetyl-Neuraminate Lyase, N-Acetyl-Neuraminate Lyase
3pvi	A	B	1.59	Pvuii Endonuclease, Pvuii Endonuclease
1f9z	A	B	1.50	Glyoxalase I, Glyoxalase I
1jr8	A	B	1.50	Erv2 Protein, Mitochondrial, Erv2 Protein, Mitochondrial
1a4i	B	A	1.50	Methylenetetrahydrofolate Dehydrogenase / Me, Methylenetetrahydrofolate Dehydrogenase / Me
1dvj	A	B	1.50	Orotidine 5'-Phosphate Decarboxylase, Orotidine 5'-Phosphate Decarboxylase
1qh4	A	B	1.41	Creatine Kinase, B Chain, Creatine Kinase, B Chain
2tnf	A	B	1.40	Tumor Necrosis Factor Alpha, Tumor Necrosis Factor Alpha
3sdh	A	B	1.40	Hemoglobin I (Homodimer) (Carbon-Monoxy) – C, Hemoglobin I (Homodimer) (Carbon-Monoxy) – C
1i0h	A	B	1.35	Manganese Superoxide Dismutase Y174F Mutant, Manganese Superoxide Dismutase Y174F Mutant
1dbf	A	B	1.30	Chorismate Mutase, Chorismate Mutase
1qks	A	B	1.28	Cytochrome Cd1 Nitrite Reductase, Cytochrome Cd1 Nitrite Reductase
1hbn	B	E	1.16	Methyl-Coenzyme M Reductase I Beta Subunit, Methyl-Coenzyme M Reductase I Beta Subunit
